# 690 Early Mobilization in Children with Burns in the Pediatric Intensive Care Unit: Outcomes and Barriers

**DOI:** 10.1093/jbcr/iraf019.319

**Published:** 2025-04-01

**Authors:** Sarah Eilerman, Lauren Justice, Taylor Iske, Jason Benedict, Ben Reader, Renata Fabia, Dana Schwartz, Rajan Thakkar

**Affiliations:** Burn Center at Nationwide Children’s Hospital; Burn Center at Nationwide Children’s Hospital; Nationwide Children’s Hospital; Nationwide Children’s Hospital; Nationwide Children’s Hospital; Nationwide Children’s Hospital; Nationwide Children’s Hospital; Nationwide Children’s Hospital

## Abstract

**Introduction:**

Pediatric patients with severe burn injuries often require intensive care unit (ICU) admission. Comprehensive care for these children requires a multidisciplinary treatment team, including physical therapy (PT) and occupational therapy (OT), to support anti-deformity positioning, functional engagement, and early mobilization (EM). Although EM is becoming the standard of care in pediatric ICUs; it has not specifically been studied in the pediatric burn population, which may face unique barriers to EM participation. This study aimed to examine barriers to EM for children with burns and trends in its application.

**Methods:**

A retrospective cohort study was completed for children with burn injury admitted to the ICU at a single ABA-verified pediatric burn center from January 2015 until February 2023. Children who died during admission or had primarily inhalation injuries were excluded. Demographics, burn mechanism, total body surface area (TBSA) burn, and PT/OT visit-level data were collected for the first 14 days of ICU admission. Patient level of activity per visit was stratified into low and high activity. Documented barriers to participation in PT/OT sessions were recorded and divided into modifiable and non-modifiable categories.

**Results:**

A total of 108 children with burns received PT and/or OT services. Children had a median age of 4 (IQR: 2-9) years, a median TBSA burn of 20%, and most were white (64%) and male (66%). At time of initial PT and OT evaluation only 25% and 27% of children participated in high level of activity. However, by 14 days of ICU admission, rates of high activity increased to 52% and 59% respectively. Children with higher TBSA burn were more likely to participate in low levels of activity (p=.002). Missed therapy visits were common with 66% missing ≥1 PT session and 55% of patients missing ≥1 OT session. Involvement in testing/procedures was the most common non-modifiable barrier (54%) and nurse concern was the most common modifiable barrier (12%).

**Conclusions:**

Despite critical illness, pediatric burn patients admitted to the ICU achieved progressively higher levels of activity after serial treatment by PT and OT. Barriers to the provision of EM are prevalent, however, only some are modifiable. Future studies should focus on initiatives that reduce barriers to successful implementation of EM.

**Applicability of Research to Practice:**

Understanding the unique barriers that children with burns face to participate in EM in the ICU is key to ensuring effective strategies are implemented to overcome such barriers.

**Funding for the Study:**

N/A

